# How experimental procedures influence estimates of metacognitive ability

**DOI:** 10.1093/nc/niz009

**Published:** 2019-06-09

**Authors:** Dobromir Rahnev, Stephen M Fleming

**Affiliations:** 1 School of Psychology, Georgia Institute of Technology, 654 Cherry Str NW, Atlanta, GA, USA; 2 Wellcome Centre for Human Neuroimaging, University College London, London, UK; 3 Max Planck UCL Centre for Computational Psychiatry and Ageing Research, University College London, London, UK

**Keywords:** metacognition, confidence, staircase procedure, perceptual decision making

## Abstract

It is becoming widely appreciated that higher stimulus sensitivity trivially increases estimates of metacognitive sensitivity. Therefore, meaningful comparisons of metacognitive ability across conditions and observers necessitates equating stimulus sensitivity. To achieve this, one common approach is to use a continuous staircase that runs throughout the duration of the experiment under the assumption that this procedure has no influence on the estimated metacognitive ability. Here we critically examine this assumption. Using previously published data, we find that, compared to using a single level of stimulus contrast, staircase techniques lead to inflated estimates of metacognitive ability across a wide variety of measures including area under the type 2 ROC curve, the confidence-accuracy correlation phi, meta-*d*′, meta-*d*′*/d*′, and meta-*d*′*–d*′. Furthermore, this metacognitive inflation correlates with the degree of stimulus variability experienced by each subject. These results suggest that studies using a staircase approach are likely to report inflated estimates of metacognitive ability. Furthermore, we argue that similar inflation likely occurs in the presence of variability in task difficulty caused by other factors such as fluctuations in alertness or gradual improvement on the task. We offer practical solutions to these issues, both in the design and analysis of metacognition experiments.

## Introduction

Humans have the ability to reflect on their own decisions and judge how likely they are to be correct (Metcalfe and Shimamura 1994; Shimamura 2000; Fleming *et al.* 2012a). The ability to evaluate one’s accuracy using confidence ratings is a common measure of metacognitive ability. Individual differences in metacognitive ability have been found to correlate with brain structure and function (Fleming *et al.* 2010; Yokoyama *et al.* 2010; Baird *et al.* 2013; McCurdy *et al.* 2013; Rahnev *et al.* 2015; Allen *et al.* 2017), as well as behavioral measures such as psychiatric symptom dimensions (Rouault *et al.* 2018). Because estimation of metacognitive ability is central to understanding the psychological and neural mechanisms supporting metacognition and introspection, and may serve as a future clinical tool, it is of critical importance that these measures be as precise as possible.

A well-recognized hurdle in estimating subject-specific metacognitive ability is the influence of primary task performance on estimated metacognitive performance (Galvin *et al.* 2003; Maniscalco and Lau 2012). Specifically, higher task performance (reflected in higher “type 1” sensitivity, *d′*) has been shown to naturally lead to higher estimates of metacognitive sensitivity (Maniscalco and Lau 2014). It has been proposed that this effect can be minimized or removed by instead using measures of “metacognitive efficiency” (Fleming and Lau 2014) such as meta-*d′/d′*. We return to this issue in the Discussion section. Nevertheless, unless measures of metacognitive efficiency are employed, comparisons of metacognitive abilities of different subjects—or the metacognitive ability of the same subject in different experimental conditions—necessitate matching task performance across subjects or conditions.

In studies of metacognition of simple perceptual judgments, it is becoming common for researchers to employ a staircase procedure that adjusts the stimulus intensity throughout the experiment to achieve uniformity in stimulus sensitivity between subjects (Fleming *et al.* 2010, 2012b; Allen *et al.* 2017). This procedure is typically successful in ensuring that performance is close to a predetermined level (typically around 71% correct). Staircases are less common in other task domains such as memory, partly because they are more complex to design and implement (although see Carpenter *et al.* 2019), but we anticipate the effects of staircases on measurement of metacognition that we outline below will also manifest in other domains beyond perception.

The continuous staircase introduces stimulus variability with the tacit assumption that this variability has no direct influence on the estimated metacognitive ability. However, mixing low and high contrast stimuli may make it easier for confidence ratings to distinguish between correct and incorrect trials. To illustrate this point, consider an extreme case in which a subject is presented with an even mixture of zero-contrast trials (producing 50% correct) and full-contrast trials (producing 100% correct). The subject’s performance over all trials would thus be 75% correct, equivalent to that produced by intermediate-contrast trials. At the same time, the subject would trivially classify all zero-contrast trials with low confidence and full-contrast trials with high confidence. In contrast, categorizing intermediate-contrast trials with low and high confidence would necessarily be more difficult since all trials are similar to each other. Therefore, confidence ratings for intermediate-contrast trials would be less informative compared to the extreme mixture of zero- and full-contrast trials. In other words, an even mixture of zero- and full-contrast trials would produce the same type 1 performance as exclusively presenting intermediate-contrast trials but will likely be characterized by much better type 2 performance (confidence ratings will be more informative of accuracy). Thus, a mixture of difficult and easy trials would be expected to lead to an inflated estimate of metacognitive ability compared to the presentation of a single, intermediate difficulty level.

The notion that mixing different contrasts leads to inflated estimates of metacognitive ability has received recent empirical support. In Experiment 3 of their paper, Bang *et al.* (2019) found that mixing increasingly dissimilar contrast levels led to increasing estimates of metacognitive ability. However, Bang *et al.* only tested the influence of such mixtures on the measures meta-*d′/d′* and meta-*d′–d′*, so it is possible that the effects were partly driven by the specific assumptions inherent in these measures. More importantly, the mixtures in Bang *et al.* (2019) contained equal number of trials coming from each contrast, but staircase procedures result in a bell-shaped curve of contrast prevalence such that intermediate contrasts are presented many times and very low and high contrasts are presented fewer times. It thus remains unclear whether staircase procedures lead to a similar inflation of metacognitive ability (as compared to using a single contrast), especially across a more divergent set of measures of metacognitive ability.

Here, we tested directly whether a staircase procedure results in inflated estimates of metacognitive ability as compared to estimates of metacognitive ability based on a single difficulty level. To do so, we re-analyzed data from Fleming *et al.* (2010) in which healthy subjects gave confidence ratings following 2-interval forced choice contrast discrimination judgments. A two-down one-up staircase ran throughout the task to maintain performance at ∼71% correct. In the original paper, Fleming *et al.* reported variation in the area under the type 2 ROC (type 2 AUC) as a key measure of metacognitive ability, finding that it was uncorrelated with type 1 performance and was predicted by gray matter volume and white matter integrity in the anterior prefrontal cortex.

We computed five different common measures of metacognitive ability across three levels of stimulus variability: a single contrast level, three contiguous contrast levels, and the full range of contrast levels used in the original experiment. To anticipate, we found that increased stimulus variability led to increased estimates of metacognitive ability across all five measures. Furthermore, the level of increase between the single contrast and the full range of contrasts correlated positively with the specific amount of stimulus variability experienced by each subject. These results suggest that a mixture of difficulty levels inherent in staircase procedures indeed leads to inflated estimates of metacognitive ability.

## Methods

### Subjects

The Fleming *et al.* (2010) dataset consists of 32 subjects (17 females; range 19–37 years). Just as in the original publication, we excluded one subject from further analysis due to aberrant psychophysical task performance [their *d′* was more than three standard deviations (SDs) higher than the group mean]. The study was approved by the local Research Ethics Committee and all subjects gave written informed consent.

### Task and procedures

All experimental details are available in Fleming *et al.* (2010). Briefly, subjects completed a 2-interval forced choice in which they indicated whether the first or second temporal interval contained a pop-out Gabor patch of higher contrast. Each interval contained six Gabor patches of 20% contrast, except for a single Gabor patch of higher contrast (between 23% and 80%) that could occur in either the first or the second interval. The contrast of the pop-out Gabor patch was continually adjusted via two-down one-up staircase procedure with a fixed step size of 3% contrast to maintain ∼71% performance. The procedure decreased the stimulus contrast after every incorrect trial and increased it after two consecutive correct trials. On every trial, subjects provided a confidence rating on a six-point scale. No feedback was given until the end of the experiment. Subjects completed a practice session, followed by the main experiment consisting of six blocks of 100 trials each. There was a small practice effect such that both the mean contrast and variability of contrast values decreased after block 1. Therefore, Fleming *et al.* (2010) only analyzed the data from blocks 2–6. Here we follow the same procedure.

### Measures of metacognitive ability

To ensure that our results are not due to the idiosyncrasies of a specific measure, we computed metacognitive ability via five different measures that are common in the literature. For all five measures, the raw confidence levels from the six-point scale were used. First, we computed type 2 AUC: the area under the type 2 ROC curve, which is the same measure used in Fleming *et al.* (2010). Second, we computed phi (Kornell *et al.* 2007): the Pearson correlation between confidence and accuracy. Finally, we computed three measures derived from the work of Maniscalco and Lau (2012): meta-*d*′, meta-*d*′/*d*′, and meta-*d*′–*d*′. These five measures each invoke different sets of assumptions (Fleming and Lau 2014) and therefore we used all of them in our study (the three measures stemming from the work of Maniscalco and Lau are based on the same model but the different ways of normalizing meta-*d*′ come with different inherent assumptions about the best way to quantify the underlying metacognitive ability).

To compute type 2 AUC, we used the same steps as described in Fleming *et al.* (2010). Briefly, for each confidence criterion, we computed type 2 hit and false alarm rates, where type 2 hits are defined as high confidence correct responses, type 2 misses as low confidence correct responses, type 2 false alarms as high confidence incorrect responses, and type 2 correct rejections as low confidence incorrect responses. We constructed a type 2 ROC curve based on the type 2 hit and false alarm rates estimated at each level of confidence, and the area under that curve produced the type 2 AUC measure.

To compute phi, we created an accuracy variable where we coded each correct response as “1” and each incorrect response as “0.” The measure phi was then computed as the across-trial Pearson correlation of this accuracy variable and the confidence ratings produced on different trials.

Finally, to compute meta-*d*′, meta-*d*′/*d*′, and meta-*d*′–*d*′, we used the code provided by Maniscalco and Lau (2012). The measure meta-*d′* is computed by determining the *d′* value that would produce the observed type 2 hit and false alarm rates under a signal detection theory model which assumes perfect metacognition. The additional measures meta-*d′/d′* and meta-*d′–d′* were obtained by normalizing meta-*d′* by *d′* via division or subtraction, respectively.

### Analyses

To compare how estimates of metacognitive ability change systematically across different levels of stimulus variability, we created three subsets of the data with increasing amount of stimulus variability.

First, for each subject, we found the contrast level that the subject experienced most frequently. The most common contrast was presented on average 122.1 times per subject (SD = 19.7). We then computed all five measures of metacognition based only on the subset of trials in which subjects experienced this contrast value. We call this the “1-contrast” condition to indicate that it consisted of trials of a single contrast.

Second, for each subject, we determined a set of three consecutive contrast values such that the most common contrast (used in the first condition) was always the middle contrast. In other words, in this condition we analyzed the most common contrast together with its two “flanking” contrasts, which were the closest contrasts to the most common one and differed from it by a single step size of the staircasing procedure. This condition included on average 320.3 trials per subject (SD = 45). Each measure of metacognitive ability was then computed for all trials within this subset. We call this the “3-contrast” condition to indicate that it consisted of trials of three contiguous contrast values.

Third, we computed all metacognition measures based on all 500 trials experienced by each subject irrespective of contrast. Note that this is how the data were analyzed in Fleming *et al.* (2010). We call this the “all-contrast” condition to indicate that it consisted of all contrast values used in the experiment.

We compared the estimated measures in each of these three conditions using one-way repeated measures ANOVAs (with factor condition) and followed up with the paired *t*-tests. We further investigated whether the differences in estimated metacognitive ability between conditions could be due to the different number of trials that went into each of the three conditions. For each subject, we considered the pool of trials in the 3- and all-contrast conditions and drew a random sample of these trials such that the size of the sample was equal to the number of trials in the 1-contrast condition for that subject. We repeated the procedure 100 times and re-did the analyses above using the averaged metacognitive scores across all 100 samples in the 3- and all-contrast conditions.

Finally, we correlated the amount of stimulus variability experienced by each subject with the amount of metacognitive inflation caused by the use of the staircase. Stimulus variability was defined as the SD of the contrast values experienced by each subject normalized by the average contrast value. Metacognitive inflation was defined separately for each measure of metacognitive ability as the difference in the estimated value in the all-contrast and the 1-contrast conditions:

$$M_{\text{inflation}}=M_{\text{all}-\text{contrast}}-M_{1-\text{contrast}}$$

where *M* refers to a particular measure of metacognitive ability. Finally, we performed a Pearson correlation to compute the level of association between stimulus variability and metacognitive inflation across subjects.

### Data and code

Processed data, together with MATLAB codes that generate all figures and statistical results, are freely available at: https://github.com/DobyRahnev/staircase_meta_inflation.

## Results

**Figure 1.**
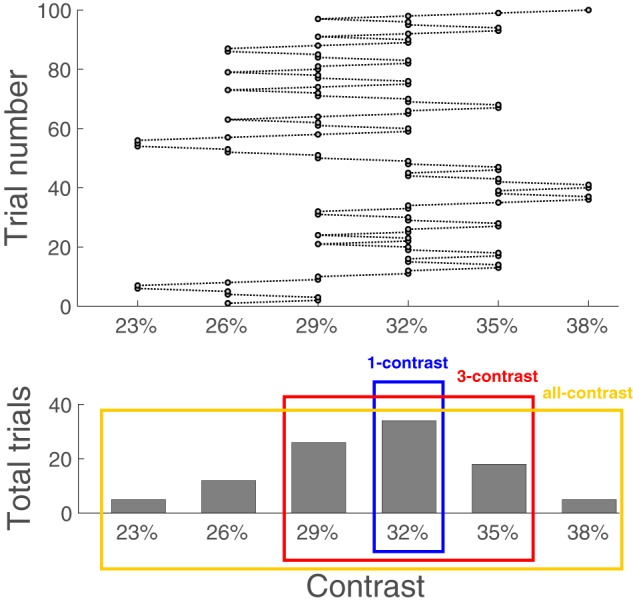
Defining three conditions with increasing level of stimulus variability. The upper panel shows the contrast values experienced by an example subject during one block of 100 trials. The lower panel shows the total number of trials in which each contrast level was presented. The three colored rectangles depict how each condition was defined. The “1-contrast” condition (blue rectangle) consisted of the most frequent contrast only. The “3-contrast” condition (red rectangle) consisted of the most frequent contrast, as well as its two flanking contrasts. Finally, the “all-contrast” condition (yellow rectangle) consisted of all contrasts experienced by the subject. Note that these three conditions were defined for each subject separately after aggregating the data from all five blocks experienced by that subject.

We investigated whether increased stimulus variability caused by continuously adjusting stimulus contrast affects various measures of metacognitive ability. To do so, we defined three conditions with increasing stimulus variability: (i) “1-contrast” condition, in which we analyzed only the contrast experienced most frequently by each subject, (ii) “3-contrast” condition, in which we analyzed the most frequent contrast together with its two flanking contrasts, and (iii) “all-contrast” condition, in which all stimulus contrasts were analyzed (a graphical depiction is provided in Fig. 1).

**Figure 2.**
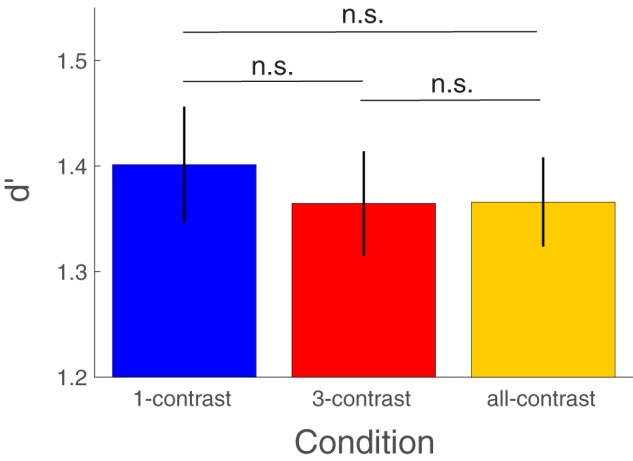
Stimulus sensitivity *d′* for each stimulus variability condition. The 1-contrast, 3-contrasts, and all-contrast conditions were well matched on stimulus sensitivity (*d′*) with all pairwise comparisons failing to reach significance (all *P *>* *0.2). Error bars depict standard error of the mean. n.s., not significant.

We first compared how stimulus sensitivity, *d′*, was affected by the three stimulus variability conditions. A one-way repeated measures ANOVA showed no significant differences between conditions [*F*(2, 60) = 0.71, *P* = 0.49; Fig. 2, individual data and observation densities in the form of raincloud plots (Allen *et al.* 2018) are shown in Supplementary Fig. S1]. Furthermore, the paired *t*-tests did not show significant difference between any pair of conditions [1-contrast vs. 3-contrast: *t*(30) = 1.07, *P* = 0.29; 1-contrast vs. all-contrast: *t*(30)  = 0.81, *P* = 0.42; 3-contrast vs. all-contrast: *t*(30) = −0.05, *P* = 0.96]. In addition, the average *d′* across the three conditions did not correlate across subjects with either the experienced stimulus variability (*r* = 0.07, *P* = 0.7) or the pairwise differences between the metacognitive scores for the all- and 1-contrast conditions (type 2 AUC: *r* = −0.2, *P* = 0.28; phi: *r* = −0.18, *P* = 0.32; meta-*d′*: *r* = −0.19, *P* = 0.34; meta-*d′/d′*: *r* = −0.06, *P* = 0.76; meta-*d′–d′*: *r* = 0.01, *P* = 0.95). These results suggest that the three conditions were well matched on primary task performance and any differences in metacognitive ability cannot be attributed to differences in task performance.

**Figure 3.**
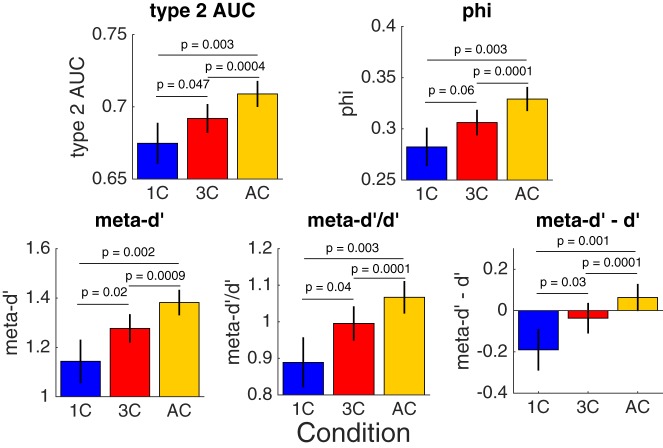
Metacognitive ability estimated for each stimulus variability condition. Estimated metacognitive ability monotonically increased between the 1-contrast, 3-contrast, and all-contrast conditions. The *P-*values report the result of the paired *t*-tests between conditions. Error bars depict standard error of the mean. 1 C, 1-contrast condition; 3 C, 3-contrast condition; AC, all-contrast condition.

We next turned to the critical question regarding the effect of stimulus variability on the estimated metacognitive ability. To avoid biasing our results by the idiosyncrasies of any individual measure, we examined the effect of stimulus variability on five different measures of metacognitive ability: type 2 AUC, phi, meta-*d′*, meta-*d′/d′*, and meta-*d′–d′* (see Methods section). For each measure, we conducted a one-way repeated measures ANOVA to check for differences between the different conditions. We found that in all cases the estimated metacognitive ability was significantly influenced by the level of stimulus variability [type 2 AUC: *F*(2, 60) = 8.66, *P* = 0.0005; phi: *F*(2, 60) = 8.38, *P* = 0.0006; meta-*d′*: *F*(2, 60) = 9.68, *P* = 0.0002; meta-*d′/d′*: *F*(2, 60) = 8.45, *P* = 0.0006; meta-*d′–d′*: *F*(2, 60) = 9.61, *P* = 0.0002; Fig. 3, individual data and observation densities in the form of raincloud plots (Allen *et al.* 2018) are shown in Supplementary Fig. S2].

To understand the nature of the observed differences in metacognition, we compared different pairs of conditions using the paired *t*-tests. First, we compared the 1-contrast and all-contrast conditions. We found that the all-contrast condition led to significantly higher estimates of metacognitive ability for all five measures [type 2 AUC: difference = 0.034; *t*(30) = 3.18, *P* = 0.003, Cohen’s *d* = 0.57; phi: difference = 0.047; *t*(30) = 3.18, *P* = 0.003, Cohen’s *d* = 0.57; meta-*d′*: difference = 0.238; *t*(30) = 3.38, *P* = 0.002, Cohen’s *d* = 0.61; meta-*d′/d′*: difference = 0.178; *t*(30) = 3.3, *P* = 0.003, Cohen’s *d* = 0.59; meta-*d′–d′*: difference = 0.253; *t*(30) = 3.51, *P* = 0.001, Cohen’s *d* = 0.63]. Comparing the 3-contrast and all-contrast conditions produced smaller differences in estimated metacognitive ability (see Fig. 3) but all these differences were nevertheless highly significant (*P *<* *0.0009 and Cohen’s *d *>* *0.66 for all measures; detailed statistical results are reported in Supplementary Results). The fact that the differences between the 3-contrast and all-contrast conditions produced consistently lower *P*-values than the difference between the 1-contrast and all-contrast condition is likely due to the smaller number of trials in the 1-contrast condition (which led to higher variability in estimated metacognitive ability, see Supplementary Fig. S2). Finally, the comparison between the 1-contrast and 3-contrast conditions was significant for four of the five measures [type 2 AUC: *t*(30) = 2.07, *P* = 0.047; meta-*d′*: *t*(30) = 2.4, *P* = 0.022; meta-*d′/d′*: *t*(30) = 2.12, *P* = 0.042; meta-*d′–d′*: *t*(30) = 2.3, *P* = 0.028] and non-significant for one measure [phi: *t*(30) = 1.95, *P* = 0.061].

One difference between the three conditions is that they were not matched for number of trials: the 1-contrast condition included on average 122 trials per subject, the 3-contrast condition included on average 320 trials per subject, and the all-contrast condition always included all 500 trials per subject. Previous research has suggested that the number of trials can bias measures of metacognitive ability (Fleming 2017). Therefore, in a control analysis, we created 100 random samples from the 3- and all-contrast conditions such that each sample contained the same number of trials as the 1-contrast condition for that subject. We then averaged the metacognitive scores obtained from these 100 samples and repeated the above analyses (Supplementary Fig. S3). We obtained very similar results: all five ANOVAs remained significant (all *P *<* *0.0015; detailed statistical results are reported in Supplementary Results) and so did all five pairwise comparisons between the 1- and all-contrast conditions (all *P *<* *0.0065; detailed statistical results are reported in Supplementary Results). Thus, the increase in the estimated metacognitive ability with increasing contrast ranges cannot be explained by the difference in number of trials between the conditions.

These results establish that conditions that feature increasingly divergent contrast levels result in increasingly higher estimates of metacognitive ability. We quantified this metacognitive inflation as the difference in estimated metacognitive ability between the all-contrast and 1-contrast conditions (i.e. $M_{\text{inflation}}=M_{\text{all}-\text{contrast}}-M_{1-\text{contrast}}$, where *M* refers to a particular measure of metacognitive ability).

**Figure 4.**
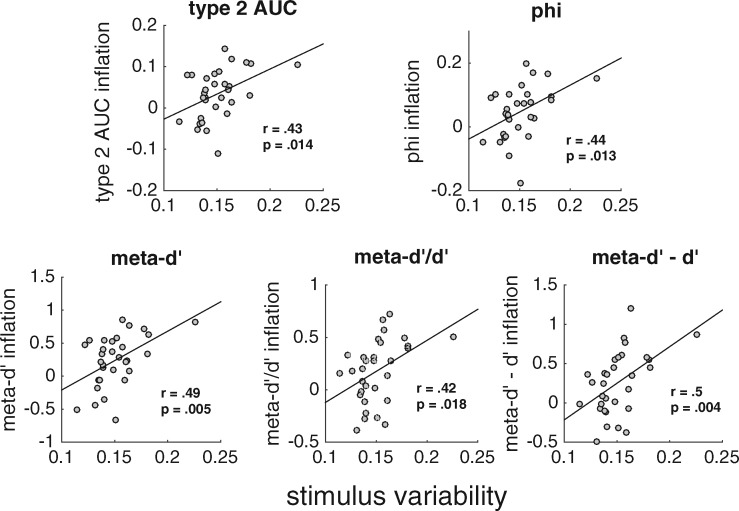
Metacognitive inflation correlates with stimulus variability experienced by each subject. For each measure of metacognitive ability, we correlated the stimulus variability experienced by each subject with the metacognitive inflation defined as the difference between the estimated metacognitive ability in the all-contrast and 1-contrast conditions. The correlation was significant for all five measures.

To identify the causes of metacognitive inflation, we next investigated whether this inflation depends on the particular level of stimulus variability experienced by a given subject. For each subject, we determined the level of experienced stimulus variability (computed as the ratio of the SD across all experienced contrasts and the average contrast). In addition, for each of the five measures, we computed metacognitive inflation as the difference in metacognitive ability between the all-contrast and 1-contrast conditions. Finally, we correlated these two quantities separately for each of the five measures of metacognition. We found that, for each measure, metacognitive inflation was significantly correlated with stimulus variability (type 2 AUC: *r* = 0.43, *P* = 0.014; phi: *r* = 0.44, *P* = 0.013; meta-*d′*: *r* = 0.49, *P* = 0.005; meta-*d′/d′*: *r* = 0.42, *P* = 0.018; meta-*d′–d′*: *r* = 0.5, *P* = 0.004; Fig. 4; one subject exhibited slightly higher stimulus variability than the rest of the group; all correlations remain significant even after excluding this subject). In other words, the inflation of metacognitive ability was larger for subjects who experienced greater stimulus variability, further underscoring a link between these quantities.

**Figure 5.**
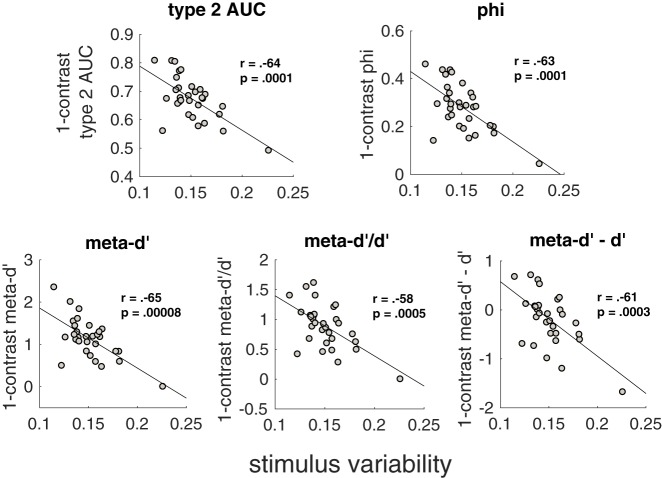
Stimulus variability is associated with metacognitive ability in the 1-contrast condition. For each measure of metacognitive ability, we correlated the stimulus variability experienced by each subject with the estimated metacognitive ability in the 1-contrast condition. The significant negative correlations may indicate that low intrinsic metacognitive ability contributes to high contrast variability in the staircase procedure.

Since stimulus variability is related to metacognitive inflation, it is important to consider what causes the difference in stimulus variability between subjects. Part of the answer is clearly random chance—the accuracy of individual responses is stochastic and therefore by chance alone some subjects will experience longer streaks of correct and incorrect responses than others, leading to higher stimulus variability. Nevertheless, it is possible that stimulus variability is at least partly driven by meaningful individual differences. One such potential difference is variability in metacognitive ability itself. Specifically, subjects with lower intrinsic metacognitive ability could be inherently more variable in their responses. If this is true, then metacognitive ability on the 1-contrast condition may correlate negatively with stimulus variability. Note that the 1-contrast condition consisted of a single contrast and therefore the metacognitive ability within that condition is statistically independent of subject-specific stimulus variability. Despite this, we observed a robust negative correlation such that higher metacognitive ability in the 1-contrast condition was associated with lower experienced stimulus variability (type 2 AUC: *r* = −0.64, *P* = 0.0001; phi: *r* = −0.63, *P* = 0.0001; meta-*d′*: *r* = −0.65, *P* = 0.00008; meta-*d′/d′*: *r* = −0.58, *P* = 0.0005; meta-*d′–d′*: *r* = −0.61, *P* = 0.0003; Fig. 5; all correlations remain significant even after again excluding the subject with highest stimulus variability). These results are consistent with the notion that the stimulus variability experienced during the staircase procedure is partly a product of intrinsic metacognitive ability (we consider other possible directions of causation in the Discussion section).

## Discussion

**Figure 6.**
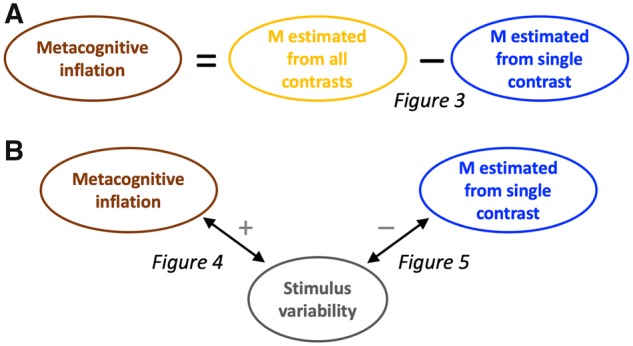
Summary of findings. (**A**) Metacognitive ability (M) was higher when estimated from trials with different contrasts (all contrasts produced by the staircase procedure) than when estimated from trials with a single contrast. This result was reported in Fig. 3. (**B**) The amount of stimulus variability across subjects correlated positively with the amount of metacognitive inflation and negatively with metacognitive ability estimated based on trials with a single contrast. These results were reported in Figs 4 and 5, respectively.

In an effort to equalize performance between different subjects or conditions, researchers are now frequently employing a staircase that continuously adjusts stimulus contrast over the duration of the experiment. Although this method is effective in ensuring that all subjects perform close to a predetermined level, it has remained unclear whether such continuous staircases affect the estimated metacognitive ability. We re-analyzed data from Fleming *et al.* (2010) in order to understand the influence of the staircase method on estimates of metacognitive ability. We obtained three primary findings, summarized graphically in Fig. 6. First, aggregating over the different contrast levels used in the staircase leads to higher estimates of metacognitive ability compared to a condition with a single contrast (Fig. 6A). We refer to such higher estimates as “metacognitive inflation.” Second, the degree of this metacognitive inflation across subjects correlates positively with the amount of stimulus variability experienced by each subject (Fig. 6B). Third, the stimulus variability experienced by each subject correlates negatively with subjects’ metacognitive ability estimated from stimuli with a single contrast (Fig. 6B). All three of these findings were robust, holding true for each of five different measures of metacognition.

### The reason for metacognitive inflation

Our most important finding was that aggregating across stimuli of different difficulty levels leads to an inflation of the estimated metacognitive ability (Figs 3 and 6A). In other words, when easier and harder trials are analyzed together, subjects’ metacognitive abilities appear to be better than they actually are. A similar effect was previously reported for the case of meta-*d′/d′* and meta-*d′–d′* in the context of mixing equal numbers of up to three different contrasts (Bang *et al.* 2019). Here, we show that this result holds across all popular measures of metacognitive ability and occurs even when the contrast mixture is bell-shaped, and itself a product of a staircase (Fig. 1).

What is the reason for metacognitive inflation? As explained in the Introduction section, mixing low and high contrast stimuli may make it easier for confidence ratings to distinguish between correct and incorrect trials. When such low and high contrast stimuli are analyzed together in the same condition, they result in higher values of estimated metacognitive ability. Another potential reason for metacognitive inflation is the presence of metacognitive noise. Metacognitive noise is noise that affects confidence estimates but not perceptual decisions (Mueller and Weidemann 2008; Jang *et al.* 2012; De Martino *et al.* 2013; Maniscalco and Lau 2016; Rahnev *et al.* 2016; van den Berg *et al.* 2017; Shekhar and Rahnev 2018). Bang *et al.* (2019) showed that combining several contrast levels in a single analysis amplifies the external stimulus variability and thus increases the sensory noise measured in the framework of signal detection theory. Furthermore, they demonstrated that increasing sensory noise leads to a decrease in both *d′* and meta-*d′* but that the decrease is larger for *d′*. Because of these differential effects on *d′* and meta-*d′*, higher sensory noise leads to increased metacognitive efficiency meta-*d′/d′* and meta-*d′–d′*. Therefore, metacognitive noise likely contributed to the increase in metacognitive efficiency scores (meta-*d′/d′* and meta-*d′–d′*) in the current analyses. However, since metacognitive noise decreases both *d′* and meta-*d′*, it should have had the opposite effect on measures of metacognitive sensitivity (i.e. meta-*d′*, type 2 AUC, and phi) (Bang *et al.* 2019). Thus, we do not consider metacognitive noise as the main contributor to the metacognitive inflation observed here.

### Metacognitive scores estimated from a single contrast are most informative about a subject’s intrinsic ability

We refer to the higher estimates of metacognitive ability in conditions that aggregate over multiple contrasts as “metacognitive inflation” (Fig. 6A). This nomenclature assumes that using a single contrast should be considered the baseline against which other conditions should be compared. What makes the single-contrast condition the best choice of baseline?

To answer this question, it is important to appreciate that the estimated metacognitive ability in each condition is a function of both a subject’s intrinsic ability and the properties of the stimulus mixture. Specifically, as more dissimilar contrasts are aggregated together, the estimated metacognitive ability would begin to reflect the properties of this mixture rather than the ability of the subject. Consider the extreme case discussed in the Introduction section where zero-contrast trials (producing chance performance) and full-contrast trials (producing 100% correct performance) are mixed. This type of extreme mixture would elicit the same pattern of response from all subjects where zero-contrast trials always receive low confidence and full-contrast trials always receive high confidence. This extreme condition would thus result in all subjects appearing to have the exact same (very high) metacognitive ability and would mask any true differences between subjects. To a smaller degree, less extreme mixtures would have the same effect, and what is worse, the estimated metacognitive ability could be contaminated by the fact that each subject experiences a different mixture. In contrast, metacognitive performance in a condition where a single difficulty level is presented should more closely track the intrinsic abilities of the subject. Therefore, we believe the single-contrast condition is most informative for revealing subjects’ intrinsic metacognitive ability. It is in this sense that we consider this condition a baseline against which other conditions should be compared.

### Recommendations for experimental design and analysis techniques

Our findings show that to accurately estimate a subject’s intrinsic metacognitive ability, an experimenter should strive to ensure that all trials experienced by the subject have the same difficulty. Continuous staircase methods violate this recommendation as they purposefully adjust the contrast of the stimulus. Furthermore, staircases with relatively large step sizes are likely to be especially problematic, whereas staircases with very small step sizes may only induce negligible stimulus variability.

Other experimental designs feature the presentation of a few predetermined difficulty levels, which are then analyzed together. The current findings, together with the results of Bang *et al.* (2018), suggest that this practice can be expected to also lead to inflated estimates of metacognitive ability. Therefore, to obtain unbiased estimates, each contrast should be analyzed separately (the estimates obtained from each difficulty level could then be averaged together in order to obtain a single metacognitive score per subject).

A related problem arises from the use of stimuli that inherently vary in difficulty. For example, many neuroimaging studies employ stimulus categories such as faces, scenes, or objects. It is virtually impossible to ensure that different exemplars of these categories are matched on difficulty (e.g. it is likely that some faces are easier to recognize than others regardless of whether they are embedded in noise, backward-masked, etc.). Therefore, the use of such categories is likely to also result in metacognitive inflation compared to designs that use stimuli—such as Gabor patches, moving dots, etc.—with small or negligible differences between different exemplars.

Even when researchers include only a single contrast level of a stimulus category with individual exemplars that do not significantly vary in difficulty, it is likely that the difficulty levels experienced by a particular subject would still vary. This could happen due to learning (Watanabe and Sasaki 2015; Dosher and Lu 2017) or variations of attention over the course of the experiment (Maniscalco *et al.* 2017). Since both of these effects are difficult to avoid completely, it is likely that every experiment leads to at least a slight inflation of the estimated metacognitive ability and that subjects with larger learning effects or more attentional fluctuations show larger metacognitive inflation. In some cases, it may in fact be beneficial to include a continuous staircase with a very small step size in order to counteract effects of learning or distraction, especially when testing patient populations where extensive periods of training may be impractical. Nevertheless, we suspect that in most cases even such “minimal” staircases would result in higher, rather than lower, overall variability in task difficulty. These considerations highlight the need for great care in experimental design, which should be optimized for the specific constraints of each experiment.

In general, we recommend designing experiments in ways that minimize extraneous influences on the estimated metacognitive ability. Most critically, we suggest the gold standard is the use of a single level of contrast. If several levels of contrast are included, then, ideally, metacognitive ability would be computed for each of them separately and the results averaged. However, determining in advance the level of contrast that would produce a desired level of performance is always subject to error (Watson and Pelli 1983), and may fail to precisely equate subjects on primary task performance. Nevertheless, in such cases, employing measures of metacognitive efficiency such as meta-*d′/d′* may be preferable to staircases in allowing a meaningful comparison of metacognitive ability between subjects with varying levels of primary task performance (Maniscalco and Lau 2012, 2014). If a continuous staircase running throughout the experiment is still judged to be needed, the variability of stimulus contrast can be estimated for each subject and included as a covariate in subsequent analyses as an approximate way of diminishing its influence. Note, however, that the metacognitive inflation introduced by combining different contrasts is unlikely to be a perfectly linear function of stimulus variability, and is therefore unlikely to be fully accounted for by a simple regression. In addition, researchers should also attempt to minimize the learning that takes place over the course of an experiment by providing subjects with an extended training period. Subjects should also be given sufficient rest throughout the experiment to minimize performance fluctuations due to fatigue and attentional lapses.

Finally, we make all of these recommendations in the context of estimating subjects’ metacognitive abilities, but the same recommendations generalize to other task domains and dependent variables. That is, for most (perhaps all) psychophysical quantities of interest, extreme mixtures of difficulty levels would necessarily elicit a pattern of responses that reflect stimulus properties rather than an individual’s ability or bias. Thus, the use of a single difficulty level may provide benefits in precision across multiple dependent measures.

### Implications for previous findings using the staircase method

A number of studies have used the continuous staircase method to correlate metacognitive ability with various quantities of interest, such as brain structure and function (e.g. Fleming *et al.* 2010, 2012b; Allen *et al.* 2017). How should we interpret these findings in light of the current results?

Following the logic outlined in Fig. 6A, suppose that previous studies set out to measure the correlation between a certain quantity (e.g. brain structure, *S*) and metacognitive ability (*M*). However, instead they measure the correlation between *S* and *M *+* I*, where *I* is the metacognitive inflation that resulted from the staircase procedure. The critical question is whether $\mathrm{corr}\left(S,M\right)$ and $\mathrm{corr}\left(S,M+I\right)$ are significantly different from each other. This question is impossible to answer in the abstract as it depends on the specifics of how much inflation has been induced, and we do not have an estimate of *I* that is independent of *M* (since *I* is operationalized as $I=M_{\text{all}-\text{contrast}}-M_{1-\text{contrast}}$). However, it is likely that *I* depends on stimulus variability, Var, which we can measure. In fact, in the current dataset, we show that Var correlates positively with *I* (Fig. 6B, left). In addition, Var correlates negatively with *M* (Fig. 6B, right), which means that adding *M* and *I* together acts to add noise to *M*. Therefore, the most likely influence of analyzing $\mathrm{corr}\left(S,M+I\right)$ is to weaken correlations that would have been obtained by analyzing $\mathrm{corr}\left(S,M\right)$, suggesting that the findings of prior studies are likely to only become stronger with the use of a constant-stimulus design.

On a practical level, as described above, one simple method for controlling for possible inflation effects is to enter staircase variability as a covariate in analyses linking *M* to *S*. Fleming *et al.* (2010) report this analysis in the supplement of their paper, showing that individual differences in brain structure (prefrontal gray matter volume and white matter integrity) remained significantly correlated with type 2 AUC after controlling for the mean and variability (SD) in the staircase, and staircase variability was not itself a predictor of brain structure in whole-brain analyses. The current study provides a principled rationale for the routine application of such control analyses in future studies that employ a staircase procedure, and suggests that cleaner results may be obtained with constant-stimulus designs.

### How should we interpret the negative correlation between metacognitive ability and stimulus variability?

We found that the stimulus variability experienced by each subject was negatively correlated with metacognitive ability, even when that ability was estimated from stimuli presented with a single level of contrast (Figs 5 and 6B). These results are consistent with the possibility that lower metacognitive ability causes more variable performance on the primary task. However, it is also possible that the direction of causality runs in the opposite direction, with higher stimulus variability decreasing metacognitive performance even in the single-contrast condition. The mechanism for this effect could be based on the fact that within the context of the current experiment, the single-contrast condition is embedded within trials of variable contrast, which could have influenced subjects’ responses in that condition. Indeed, confidence ratings exhibit suboptimal serial dependence (Rahnev *et al.* 2015; Rahnev and Denison 2018), such that subjects may use different confidence criteria in the single-contrast condition after having experienced either low or high contrast in the previous few trials. Such variability of confidence criteria would in turn result in decreased metacognitive ability (Shekhar and Rahnev 2018), thus potentially driving the observed negative correlation.

Further research is needed in order to distinguish between these two possible causes of the observed negative correlation between metacognitive ability and stimulus variability. Specifically, it would be important to test the correlation between the variability of contrasts in a staircase and the metacognitive ability estimated from a separate block of trials with a single contrast. If lower metacognitive ability can indeed be causally linked to more variable task performance, this finding would suggest that people’s metacognitive ability influences not only their confidence ratings but also affects the stability of primary task performance.

## Conclusion

We have demonstrated that combining multiple difficulty levels in a single analysis leads to an inflation of estimated metacognitive ability, relative to a constant-stimulus design. While this effect can never be avoided completely, researchers should be aware of it and attempt to minimize it whenever possible. We suggest that researchers aim for a constant-stimulus design, and where this is not possible, analyze each difficulty level separately and avoid aggregating over different difficulty levels. We believe that such design considerations will provide more robust measures of metacognition, and in turn drive forward our understanding of the psychological and neural mechanisms supporting metacognition and introspection.

## Supplementary Material

niz009_Supplementary_Data
