# How experimental procedures influence estimates of metacognitive ability

**DOI:** 10.1093/nc/niz010

**Published:** 2019-07-15

**Authors:** Dobromir Rahnev, Stephen M Fleming

**Affiliations:** 1 School of Psychology, Georgia Institute of Technology, 654 Cherry Str NW, Atlanta, GA, USA; 2 Wellcome Centre for Human Neuroimaging, University College London, London, UK; 3 Max Planck UCL Centre for Computational Psychiatry and Ageing Research, University College London, London, UK

*Neuroscience of Consciousness*, Volume 2019, Issue 1, 2019, https://doi.org/10.1093/nc/niz009

In the original version of this article, the section ‘The reason for metacognitive inflation’ contained a small error. The corrected sentence reads as follows:

Therefore, metacognitive noise likely contributed to the increase in metacognitive efficiency scores (meta-d′/d′ and meta-d′–d′) in the current analyses but not to the increase in metacognitive sensitivity scores (meta-d′, type 2 AUC, and phi) (Bang et al. 2019).

The authors would like to apologize for any confusion this may have caused.

