# Supplementary material for: How experimental procedures influence estimates of metacognitive ability
Source: Neurosci Conscious. 2019 Jun 9;2019(1):niz009. doi: 10.1093/nc/niz009 (PMC6556214; doi:10.1093/nc/niz009)
Supplement: niz009_Supplementary_Data [file niz009_supplementary_data.docx]

**Supplementary Material for Rahnev & Fleming “How experimental procedures influence estimates of metacognitive ability”**

**Supplementary Results**

Here we report in detail the results of several statistical tests that were only summarized in the main text. First, the Results section in the main paper reported that the metacognitive scores were significantly higher in the all-contrast compared to the 3-contrast condition. Indeed, this was true for each of the five measures of metacognition (*type 2 AUC*: difference = .017; t(30) = 3.996, *p* = .0004, Cohen’s d = .72; *phi*: difference = .023; t(30) = 4.56, *p* = .00008, Cohen’s d = .82; *meta-d’*: difference = .11; t(30) = 3.69, *p* = .0009, Cohen’s d = .66; *meta-d’/d’*: difference = .07; t(30) = 4.43, *p* = .0001, Cohen’s d = .79; *meta-d’–d’*: difference = .1; t(30) = 4.35, *p* = .0001, Cohen’s d = .78).

Second, the Results section in the main paper reported that the control analyses in which we equated the different conditions on number of trials resulted in similar results as our main analyses. Specifically, all five ANOVAs remained significant (*type 2 AUC*: F(2,60) = 7.37, *p* = .001; *phi*: F(2,60) = 8.07, *p* = .0008; *meta-d’*: F(2,60) = 7.82, *p* = .001; *meta-d’/d’*: F(2,60) = 10.39, *p* = .0001; *meta-d’–d’*: F(2,60) = 8.31, *p* = .0007). In addition, all five pairwise comparisons between the 1- and all-contrast conditions remained significant too (*type 2 AUC*: t(30) = 2.93, *p* = .006, Cohen’s d = .53; *phi*: t(30) = 3.09, *p* = .004, Cohen’s d = .55; *meta-d’*: t(30) = 3.03, *p* = .005, Cohen’s d = .54; *meta-d’/d’*: t(30) = 3.57, *p* = .001, Cohen’s d = .64; *meta-d’–d’*: t(30) = 3.23, *p* = .003, Cohen’s d = .58).

**Supplementary Figures**

**Supplementary Figure 1. Stimulus sensitivity (*d’*) for each stimulus variability condition**. This figure re-plots the data from Figure 2 to provide more information about the underlying data. The figure is an adaptation on the raincloud plots introduced by Allen et al. (2019). The upper part of the figure depicts the densities of observations for each condition. The lower part of the figure plots the individual observations and corresponding boxplots.

**Supplementary Figure 2. Metacognitive ability estimated for each stimulus variability condition.** This figure re-plots the data from Figure 3 to provide more information about the underlying data. The figure is an adaptation on the raincloud plots introduced by Allen et al. (2019). The upper part of each subplot depicts the densities of observations for each condition. The lower part of each subplot depicts the individual observations and corresponding boxplots.

**Supplementary Figure 3. Metacognitive ability estimated for each stimulus variability condition while controlling for the number of trials per condition**. This figure shows the results of the control analysis, in which we created 100 random samples from the 3- and all-contrast conditions such that each sample contained the same number of trials as the 1-contrast condition for that subject. We then averaged the metacognitive scores obtained from these 100 samples. As can be seen from the figure, the results were very similar to what we obtained in our main analyses reported in Figure 3. As in Figure 3, the *p* values report the result of paired t-tests between different conditions and error bars depict standard error of the mean. 1C, 1-contrast condition; 3C, 3-contrast condition; AC, all-contrast condition.
